# Preconcentration and Determination of Psychotropic Drugs in Urine Samples by Ion Mobility Spectrometry with Electrospray Ionization Coupling On-Line Single-Drop Liquid-Liquid-Liquid Microextraction

**DOI:** 10.1155/2019/8561801

**Published:** 2019-11-13

**Authors:** Shu Li

**Affiliations:** National Institutes for Food and Drug Control, Institute for Medical Devices Control, No. 29, HuaTuo Road, Daxing District, Beijing 102629, China

## Abstract

A method for analysis of psychotropic drugs in urine is investigated using a novel single-drop liquid-liquid-liquid microextraction (SDLLLME) apparatus as an electrospray emitter in ion mobility spectrometry (IMS). In this method, ketamine and pethidine are back-extracted into the acceptor phase (water and methanol) from the water and methanol immiscible organic phase. Sensitivity of extraction is improved as it does not require further methanol-adding procedure. Factors affecting the extraction of psychotropic drugs are characterized, including organic solvent type, extraction time, and concentration of NaOH/Ac in the donor/acceptor phase. The best extraction sensitivity is obtained with 600 *μ*L dodecane as the organic phase, 30 minutes extraction time, and 10 mL donor phase with 0.01 M sodium hydroxide (NaOH) and 3 *μ*L acceptor phase with 0.5 M acetic acid (Ac). Using this method, the two analytes can be extracted and analyzed simultaneously, showing this method is valuable for field application.

## 1. Introduction

Psychotropic drugs are mind-altering substances affecting the central nervous system that can lead to a variety of changes in consciousness, behaviour, mood, and perception [1]. Under medical supervision, they can be of great help to patients. However, nonmedical use of these drugs has been increasing and becoming a medical and social issue of many countries. Liquid chromatography-mass spectrometry has been the primary method for analysing psychotropic drugs. However, the increasing demands for this examination require the capability of direct detection and quantitative analysis. Ion mobility spectroscopy (IMS) meets these requirements. It is a gas-phase electrophoretic method in which ions are separated on the basis of their relative mobility. In the past two decades, it has been proven a reliable method for rapid and sensitive detection of trace substances, including residues and breakdown products of explosives [2, 3] and chemical warfare agents [4–6], as well as illicit drugs [7–9].

For psychotropic drug determination, biological samples include urine, blood, hair, saliva, and so on. Different biological samples can provide different information. Drug excretion in vivo is mainly in the form of urine. Drugs in urine exist in the form of prototype, conjugate, or metabolite. Therefore, human urine is the most valuable sample for drug abuse analysis because of its high drug content, convenient collection, and long existence in the human body. Compared with urine, the blood drug concentration is low and the material and preservation are inconvenient. Drugs also accumulate in hair, which makes hair become the sample for drug abuse analysis. Hair analysis can reflect drug abuse for a long time. However, the general content of drugs in hair is low, the exogenous pollution and endogenous interference are more serious compared with other biological samples, and the pretreatment steps such as collection, washing, and extraction are cumbersome. Saliva analysis is more easily accepted by patients. Saliva analysis is not limited by the site and gender. However, saliva analysis is suffered from contaminating by food, chewing gum, cigarettes, etc., which may cause interferences. For reliable result, sample cleanup and enrichment prior to detection are important to avoid interfering substances. Solid-phase microextraction (SPME) [10] and liquid-phase microextraction [11] (LPME) are the most commonly used techniques that can extract analytes from liquid and gaseous matrices to chromatographic detection. They have several advantages over the conventional techniques of sample treatment, liquid-liquid extraction (LLP) or solid-phase extraction (SPE), such as solvent-free or minimal consumption of hazardous solvents, minimal amount of sample analyzed, and higher enrichment factor.

Liquid-liquid-liquid microextraction (LLLME), introduced by Ma and Cantwell in 1998 [12] and the Pedersen-Bjergaard group in 1999 [13], is a three-phase LPME method. In this method, the analytes are extracted from an aqueous sample matrix (donor phase) into the organic solvent (organic phase) and then back-extracted into another aqueous solution (acceptor phase). Based on the organic phase and acceptor phase interface, there are two kinds of LLLME. The first kind is that the organic phase lies out on top of the donor phase as a solvent membrane, and the acceptor phase is suspended as an immiscible single drop in the organic phase (SDLLLME) [12]. The second kind is that a hollow fiber (HF-LLLME) [13] is immersed into the donor phase completely with the organic phase immobilized into the porousness of the fiber, and the acceptor phase filled inside of the fiber.

Ion mobility spectroscopy (IMS) is an analytical method that is ideally suitable for the detection of trace amounts of the specific compound in a gaseous mixture. Intrinsically, IMS is an appropriate method in areas where real-time analyses are necessary, field applications are needed, and most samples are expected to be negative [14]. Therefore, IMS is well suited for large-scale, trace-level investigation and monitoring programs where the sophisticated laboratory instrument is not available. Electrospray ionization (ESI), first introduced by Dole et al. and later developed by Fenn et al. [15] as a soft ionization source for MS, has been successfully combined with IMS by Shumate and Hill. Using this hyphenated method, a liquid sample can be transferred directly into IMS as gas-phase ions and then separated and detected by the mobility spectrometric method. Several groups have reported the ESI-IMS in analysis of drugs. Saraji et al. [16] combined hollow fiber liquid-liquid-liquid microextraction and electrospray ionization-ion mobility spectrometry (ESI-IMS) for the analysis of diclofenac in biological samples. This method was also applied to analysis of urine and plasma of a healthy volunteer after oral administration of diclofenac. Peiró et al. [17] developed a method for the evaluation of the 3,4-methylenedioxypyrovalerone (MDPV) in oral fluids using IMS with liquid-liquid microextraction. Armenta et al. [18] developed two different strategies for determining abuse of ecstasy based on the analysis of saliva by ion mobility spectrometry (IMS) after thermal desorption and the joint use of IMS and infrared (IR) spectroscopy after liquid-liquid microextraction (LLME).

Compared with other LPME methods, the main character of LLLME is that the analytes are finally extracted in an aqueous solution other than the organic solvent. When combining LLLME to electrospray-ion mobility spectrometry (ESI-IMS), however, this could become a drawback because the aqueous solution should be mixed with methanol or acetonitrile to enhance ionization efficiency in ESI. This mixing producer, however, dilutes the analyte concentration and consequently spoils the detection limit. To overcome this drawback, the new concept of LLLME based on two immiscible organic solvents was used to extract analytes from aqueous samples to a mixture of methanol and water. The performance of this strategy was compared with LLLME aqueous extraction which subsequently diluted using methanol. Meanwhile, the extraction needle is modified to be directly used as an electrospray emitter to reduce the sample lost during the transfer. Different factors (organic solvent type, extraction time, composition of donor, and acceptor solutions) affecting the extraction efficiency are investigated. The optimized conditions are applied to human urine analysis.

## 2. Materials and Methods

### 2.1. Extraction Apparatus and LLLME Procedure

To compare with the original water immiscible organic solvents mode (WIM-LLLME), the two immiscible organic solvents mode (TIM-LLLME) should share with the same extraction apparatus. However, in TIM, methanol : water (9 : 1) with acetic acid was used as the accepter phase. Water and methanol immiscible alkanes were use as the organic phase. In WIM, the accepter phase contains only water with acetic acid. Water immiscible organic solvents were used as the organic phase. The accepter phase droplet after TIM can be directly electrosprayed for IMS analysis. The droplet after WIM should be mixed with methanol.

**Figure 1:**
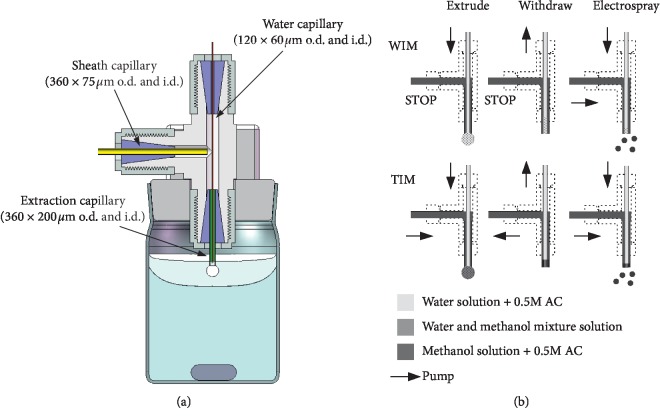
Schematic of the SDLLLME apparatus (a) and pump strategy (b) of the two modes in different steps.

For direct electrospray analysis in both TIM and WIM, the extraction apparatus is fabricated to allow methanol-adding directly inside it. The apparatus setup is schematically shown in Figure 1. The extraction apparatus was built following the sheath flow design that is always used in the capillary electrophoresis-electrospray ionization interface. It is assembled using the stainless-steel Tee fitting (Upchurch Scientific) with a coaxial fused-silica water capillary (120 × 60 *μ*m o.d. and i.d.) through the 200 *μ*m orifice of the Tee fitting and the extraction capillary (360 × 200 *μ*m o.d. and i.d.). The smaller difference between the external diameter (120 *μ*m) of the water capillary and the internal diameter of the extraction capillary (200 *μ*m) is expected to provide the sheath flow (methanol) to modify the composition of the accepter phase during the electrospray procedure. The pump strategy is also illustrated in Figure 1. In WIM, at the steps of extrude and withdraw, only water capillary is forced by the syringe pump to guarantee water immiscible extraction. At the electrospray step, water capillary and sheath capillary (containing methanol) are forced together; therefore, the mix process can be achieved at the tips of the extraction capillary. In TIM, two capillaries can be forced together at all three steps.

The overall extraction procedure is in accordance with the literature with some modifications [19]. First, analytes aqueous solution (donor phase) pH is adjusted to the desired value by adding an appropriate amount of 1 M NaOH to the solution. Then, 10 mL donor phase and 750 *μ*L of organic phase are added to the extraction flask subsequently by a micropipette sampler. After mixing, the extraction flask was sealed with the Polytetrafluoroethylene (PTFE) plug and mounted on a magnetic stirrer (Corning Inc., Model PC-220) and stirred for some time at the moderate speed (800 rpm) by the stirring bar (length: 20 mm; diameter: 6 mm) for accelerating the organic phase extraction of analytes from the donor phase. This procedure is also called pre-extraction. After the stirring was stopped, the extraction flask was resealed by another PTFE plug in which the extraction apparatus was mounted. A 3 *μ*L droplet of the accepter phase was extruded out of the capillary by a syringe pump and suspended in the layer of the organic phase. Another stirring time was used for extraction of the analytes from the organic phase. This procedure is called back-extraction. After back-extraction, the suspended droplet was withdrawn back into the extraction capillary by the syringe pump and the extraction apparatus was directly used as an electrospray emitter.

### 2.2. Drop Formation

Drop formation is a crucial step in the method development of SDLLLME. In traditional SDLLLME, a HPLC flat-cut syringe was used for suspending the acceptor drop in the organic phase during extraction and for injection after extraction. The relatively large outer diameter (700 *μ*m) and the nonwetting surface character of the syringe needle make the suspending acceptor drop stable. In our experiment, extraction capillary is used directly to suspending the drop. To guarantee the drop suspending in extraction capillary stably, careful management should be made. First, the injection and withdraw procedure are forced by the syringe pump; therefore, the drop volume could be precisely controlled; meanwhile, the drop lost due to hand tremor in man-forced injection and withdraw could be prevented. Second, to ensure the drop suspending out of the transect of the fused-silica capillary tip but not wetting of the capillary wall, the tip is first inserted vertically into boiling sulfuric acid (98%) to remove the polyimide fibril and then into hydrofluoric acid about 10 min to make the surface more hydrophobic.

### 2.3. Ion Mobility Spectrometry

The ambient pressure ion mobility spectrometer with an electrospray ionization source (ESI-IMS) used in this investigation was constructed at our laboratory. A detailed description and diagram of the IMS were described previously [20].

A voltage difference of about 2.4 kV was made between the spray emitter and drift tube to establish the electrospray process. The drift tube was assembled using stacked rings with a high voltage of +11 kV applied making a linear field of 157 V·cm^−1^ and 333 V·cm^−1^ in the desolvation region and the drift region, respectively.

The extraction apparatus was directly used as the electrospray emitter. Two independent syringe pumps were used to supply the driving force in the electrospray process making the total flow rate about 0.5 *μ*L·min^−1^.

### 2.4. Chemicals and Reagents

Ketamine (CAS #: 6740-88-1, molecular formula: C_13_H_16_ClNO) and pethidine (CAS#: 57-42-1, molecular formula: C_15_H_21_NO_2_) were obtained from China Northeast Pharmaceutical Company (Shen Yang, China). All organic solutions (dodecane, isooctane, cyclohexane, hexane, toluene, xylene, and 1-octanol) were purchased from J.T. Baker. HPLC-grade methanol (MeOH) and analytical-grade acetic acid (Ac) were purchased from Sinopharm Chemical Reagent Corp. Deionized water (18.2 MΩcm) is obtained from a water purification system (Michem, MW-D20).

Two psychotropic samples were prepared by micropipette sampling of known volumes of analytes, and then dissolving them in a 10 mL amber color volumetric flask with methanol to give analyte concentrations of 100 mgL^−1^. Samples were then stored at 4°C in a refrigerator until measurements were made. Prior to each measurement, the samples were diluted with the donor phase (pure water or urine with NaOH) to the required concentrations.

### 2.5. Urine Sample

Two urine samples were donated by a healthy male and a healthy female, respectively. The samples were stored in a refrigerator at 4°C. All samples were filtered through 0.22 *μ*m syringe filters right before use.

## 3. Results and Discussion

### 3.1. Reduced Mobility

The experimental drift time and reduced mobility constants (*K*_0_) for ketamine and pethidine are performed in our lab and are given in Table 1. Analytes are furnished as the protonated monomer (MH^+^). ESI-IMS results for the analytes are compared to the SDLLLME-IMS results and agreed well. The relative error between the reduced mobility values calculated from the spectra collected with the ESI-IMS and SDLLLME-IMS is negligible.

### 3.2. Comparison of Extraction Efficiency of Two Strategies

Water and methanol immiscible dodecane, isooctane, cyclohexane, and hexane are used in TIM and WIM as the organic phase for extraction of the ketamine and pethidine. The ion responses (peak area) are compared with toluene, xylene, and 1-octanol which could only be used in original WIM.Both pre- and back-extraction time was fixed to 40 min to allow concentration equilibrium reached in the organic and acceptor phase for two psychoactive drugs. The result is based on the average of the three measurements. 10 mL solutions with 0.5 M NaOH and 200 *μ*g·L^−1^ ketamine and pethidine were extracted with 750 *μ*L of the solvent and back-extracted by a 3 *μ*L drop with 0.5 M Ac suspended in the organic layer.

Table 2 displays the ion responses of ketamine and pethidine in the TIM and WIM with different solvents. This table shows that in TIM, the dodecane and isooctane have comparable responses and exhibit the highest sensitivity, both to ketamine and pethidine. The differences of extraction efficiency within the same mode are probably due to variations in the structure and polarity of the solvent molecule. However, compared of the extraction efficiency using the same different solvents among TIM and WIM, ketamine and pethidine sensitivity could be increased significantly if TIM could be applied. Though toluene and xylene exhibit the highest “pure” extraction efficiency (deduced from the comparison of ion responses using different solvents in WIM), subsequent methanol-adding dilutes the concentration of analytes and finally makes the ion responses worse than that in TIM. In TIM, the analytes are finally back-extracted to a mixture of water and methanol. This could be electrosprayed directly therefore avoids diluting the analytes due to methanol-adding. In summary, the dodecane in TIM exhibited the highest ion responses for ketamine and pethidine. For all further studies, the dodecane in TIM is chosen and the remaining solvents are not investigated further.

### 3.3. Extraction Time Profiles

**Figure 2:**
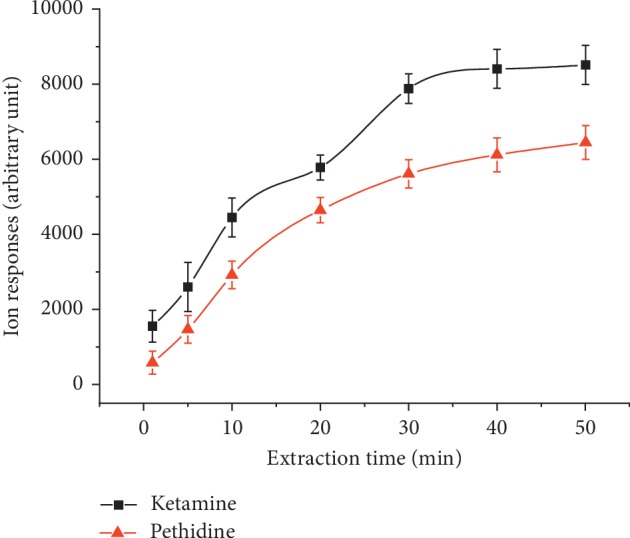
Extraction time profiles obtained for ketamine and pethidine using dodecane in TIM.

The influences of extraction time on the ion responses are investigated by comparing the response of 200 *μ*g·L^−1^ ketamine and pethidine as a function of extraction time ranging from 5 min to 50 min when other extraction conditions remain unchanged compared with extraction efficiency. Since SDME is an equilibrium extraction technique, under a given condition the maximum amount of analytes back-extracted to the accepter phase should be invariable after solution equilibrium is reached. As shown in Figure 2, the ion responses approached a plateau region around 30 min to both ketamine and pethidine. This indicates the equilibrium state between the analytes in three phases reached after 30 min of extraction. Therefore, an optimum extraction time of 30 min is chosen to achieve maximum extraction efficiency without unnecessary extending of the analysis time.

### 3.4. Composition of the Donor and the Acceptor Phases

In SDLLLME, the composition of both the donor and the acceptor phases is critical. Since ketamine and pethidine are weak basic compounds (pKa = 8.4 and 8.63), the donor phase should be sufficiently alkaline in order to deionize the analytes and consequently reduce its solubility in the donor phase. On the contrary, the acceptor phase should be sufficiently acidic that promotes the dissolution of the analytes in the acceptor phase. Meanwhile, since the acceptor phase is directly used as an electrospray solution, therefore the acidity of the acceptor phase also affects the process in ESI.

**Figure 3:**
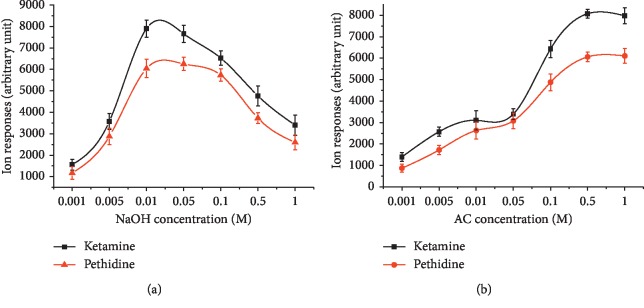
Concentration profile for NaOH (a) in the donor phase and Ac (b) in the acceptor phase.

For the donor phase, the NaOH concentration was varied between 0.001 and 1 M with an Ac concentration at 0.1 M. The experimental results in Figure 3(a) indicated that the highest ion responses were obtained at 0.01∼0.1 M NaOH. In more basic samples, a decrease in the enrichment factor was observed. This can be attributed to two reasons: One is an increase in the ionic strength of the donor phase in the presence of higher concentrations of NaOH. The other is the decrease of stability and solubility of ketamine and pethidine in the alkaline medium.

The Ac concentration of the acceptor phase was varied between 0.001 and 1 M with the NaOH concentration at 0.05 M. It was observed in Figure 3(b) that there was no IMS response of analytes when the Ac concentration was low (0.005 M). This could be explained by that the target analyte was not able to transport from the organic phase into the acceptor phase. As the Ac concentration increased from 0.01 to 0.5 M, ion response increased. Further increasing of Ac concentration above 0.5M did not affect the ion response. Therefore, 0.5 M Ac was selected as the acceptor phase for further experiment.

### 3.5. Validation of the Method

To demonstrate the feasibility of SDLLLME-IMS, the method was used for the determination of ketamine and pethidine in urine. Urine of two volunteers (10 mL, without further dilution) was used as the donor phase and spiked with different amounts of analytes. The urine was analyzed using the SDLLLME-IMS setup with the optimized condition obtained from the previous study.

**Figure 4:**
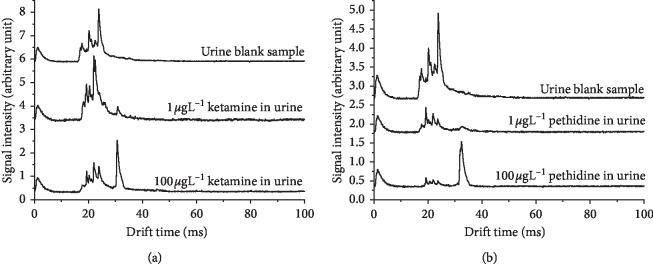
SDLLLME-IMS spectra obtained for extraction of ketamine (a) and pethidine (b) in urine at 0 *μ*g·L^−1^ (blank), 1 *μ*g·L^−1^_,_ and 100 *μ*g·L^−1^.

The SDLLLME-IMS spectra for ketamine and pethidine spiked at 1 and 100 *μ*g·L^−1^ in urine are presented in Figure 4. The solvent peaks and the analytes peaks are all seen in all the spectra. In the solvent peak region, multiple peaks are observed for each spectrum. These peaks are corresponding to the solvent ion clustered with different number of water molecules. Compared with the spectrum obtained from the pure sample, no evident differences are seen, indicating that due to the excellent sample cleanup capability of the SDME, matrix components in urine do not interfere with the detection process. The reduced mobilities of the ketamine and pethidine extracted from urine are compared with the values obtained from the pure sample and water reported in Table 1 with a difference <3%.

The analytical merits of the SDLLLME procedure was characterized in terms of linearity (linear concentration ranges, regression data, and correlation coefficient), precision (relative standard deviation, RSD), sensitivity (limits of detection, LOD), and extraction efficiency (enrichment factors and relative recovery, EF and RR). The results are summarized in Table 3.

The calibration curves are constructed for ketamine and pethidine in the urine matrix using 7 spiked levels of the drug with the concentrations ranging from 1 to 500 *μ*g·L^−1^. Each product curve is nearly linear in the concentration range between 1 and 500 *μ*g·L^−1^ (ketamine, *R*^2^ = 0.98 and pethidine, *R*^2^ = 0.99). The LODs, based on the three times noise level for ketamine and pethidine, are 0.65 and 0.91 *μ*g·L^−1^. The repeatability of the method (expressed as relative standard deviation) was evaluated at 10 *μ*g·L^−1^ (*n* = 5), resulting to be in the range from 5.7% (ketamine) to 7.6% (pethidine). The relative recovery is obtained using the following equation:

(1) $$\begin{array}{c} RR\left(\%\right)\frac{A_{1}-A_{2}}{A_{3}}\times 100, \end{array}$$

where *A*_1_, *A*_2_, and *A*_3_ are the peak areas of the extracted analyte in the spiked sample, extracted in the unspiked sample, and extracted in the spiked deionized water, respectively. For ketamine and pethidine, the relative recoveries are 88.4 and 92.7. The enrichment factors (EF) were calculated by the following equation at the 10 *μ*g·L^−1^ level (the sample concentration in the donor phase is 10 *μ*g·L^−1^):

(2) $$\begin{array}{c} \text{EF}=\frac{C_{\text{ap}}}{C_{\text{dp}}}, \end{array}$$

where *C*_ap_ and *C*_dp_ are the sample concentration in the acceptor phase and the donor phase, respectively. The sample concentration in the acceptor phase is determined by peak area using a calibration curve.

Generally, during real analysis, a urine sample containing a mixture of psychotropic drugs should be expected. Therefore, the capability of distinguishing a mixture is desirable. Therefore, ketamine and pethidine at different concentrations were mixed together and analyzed to verify the apparatus extraction and separation capability in the mixture.

**Figure 5:**
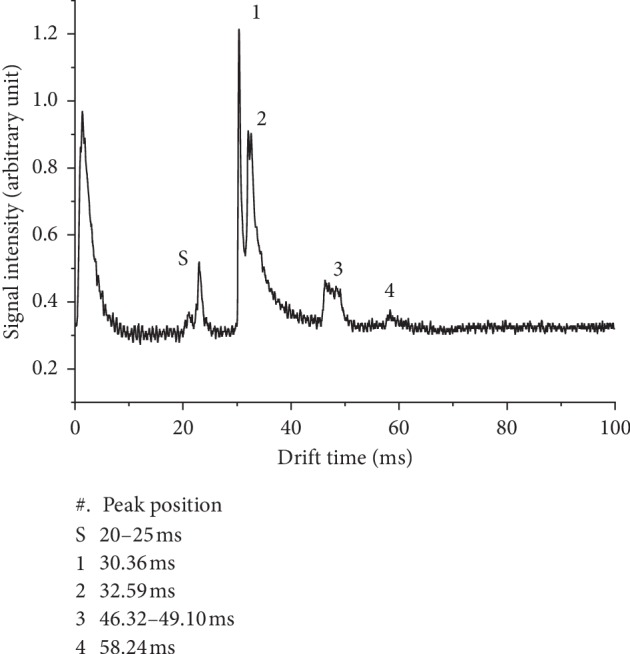
SDLLLME-IMS spectrum obtained for the extraction of a mixture of ketamine and pethidine in urine with respect to the concentration of 50 *μ*g·L^−1^. Peak 1 and Peak 2 are identified as ketamine and pethidine.

Figure 5 shows the spectrum corresponding to the mixture of ketamine and pethidine using the current SDLLLME-IMS setup. A complex spectrum of peaks was obtained as expected. Two analytes should have furnished peak; however, five peaks were found in the mixture spectrum. Reduced mobilities are a means of identifying the peaks in the SDLLLME-IMS spectra. Three of the peaks were identified through reduced mobility calculations as the solvent and monomer peaks of the analytes. Based on these results, it can be concluded that the quantitative analysis of complex urine samples is hindered. However, as a field rapid and sensitive detection method, SDLLLME-IMS can also be used as a qualitative method.

Peak 1 and Peak 2 are identified as ketamine and pethidine, respectively. The drift time and calculated reduced mobility match the value described previously. Two peaks (3 and 4) that appear at the reduced mobilities 1.35 and 1.19 cm^2^·V^−1^·s^−1^ were not identified. They were putatively identified as the ketamine and pethidine complex and fragmentation. However, mass spectroscopic identification would be needed to confirm the identities of these peaks. The relative errors between the reduced mobility values calculated from the spectra collected for the individual analyte in soil and the analyte in the mixture were negligible. The total time for the mixture analysis using the SPMEIMS was about one hour.

## 4. Conclusions

Methanol-adding procedure for electrospray can be avoided by the direct use of the extraction apparatus as an electrospray emitter, altering the widely used water immiscible organic phase into the water and methanol immiscible organic phase. Using this method, enrichment efficiency can be increased 4 times than that of original LLLME. In this work, we also characterized the influence of factors affecting the analytical signal and optimized the process. Psychotropic drugs, ketamine and pethidine, are analyzed by our system in pure water and urine, and no significant differences are observed on analysis process indicating the complex matrixes in urine have been cleaned up by our SDLLLME-IMS. Ketamine and pethidine are detected at 0.65 and 0.91 *μ*g·L^−1^ in urine. Furthermore, the methodology was validated through a mixture study using urine samples containing the two analytes. The two analytes can be extracted and analyzed simultaneously, making this method valuable for field application. In further work, we will focus on the evaluation of more complex matrixes and the potential automation of the technique.

## Figures and Tables

**Table 1:** Experimental drift time and reduced mobility constants (*K*_0_)^a^ for ketamine and pethidine, obtained for each method.

Analytes	Parameter	IMS	SDLLLME	SDLLLME
			In water	In urine
Ketamine	Drift time (ms)	30.64	30.82	30.91
	Mobility	1.47	1.46	1.46

Pethidine	Drift time (ms)	32.43	32.67	32.52
	Mobility	1.39	1.38	1.39

^a^Reduced mobility are expressed in units of cm^2^·V^−1^·s^−1^.

**Table 2:** Ion responses of ketamine and pethidine in the TIM and WIM with different solvents.

Solvent	PI	Ketamine ion responses			
		TIM		WIM	
		Mean	Standard deviation	Mean	Standard deviation
Dodecane	0.1	8540	314	2430	223
Isooctane	0.2	8170	280	2340	222
Cyclohexane	0.2	6230	261	1850	120
Hexane	0.1	5800	153	1780	220
Toluene	2.4	—	3210	136	
Xylene	2.6	—	3030	285	
1-Octanol	3.4	—	1620	158	
Dodecane	0.1	6160	233	1570	124
Isooctane	0.2	6050	227	1380	101
Cyclohexane	0.2	1080	100	370	48
Hexane	0.1	2430	281	590	149
Toluene	2.4	—	2570	199	
Xylene	2.6	—	2230	171	
1-Octanol	3.4	—	1270	192	

PI: polarity index.

**Table 3:** Calculated detection limits for ketamine and pethidine in urine.

Analytes	LOD (*μ*g·L^−1^)	Slope	Intercept	*R* ^2^	EF^a^	RR (%)^a^	RSD (%)^a,b^
Ketamine	0.65	0.0145	0.5112	0.98	108	88.4	5.26
Pethidine	0.91	0.0105	0.1549	0.99	94	92.7	7.38

^a^Enrichment factor (EF), relative recovery (RR), and relative standard deviation (RSD) were obtained at the 10 *μ*g·L^−1^ level of ketamine and pethidine in urine. ^b^*n* = 5.

## Data Availability

The data used to support the findings of this study are available from the corresponding author upon request.

## Abbreviations

SDLLLME: Single-drop liquid-liquid-liquid microextraction
IMS: Ion mobility spectrometry
WIM: Water immiscible organic solvents mode
TIM: Two immiscible organic solvents mode.
